# Evidence and importance of genetic exchange among field populations of *Trypanosoma cruzi*

**DOI:** 10.1016/j.actatropica.2015.05.007

**Published:** 2015-11

**Authors:** Louisa A. Messenger, Michael A. Miles

**Affiliations:** Department of Pathogen Molecular Biology, Faculty of Infectious Tropical Diseases, London School of Hygiene and Tropical Medicine, United Kingdom

**Keywords:** Chagas disease, *Trypanosoma cruzi*, Genetic exchange, Recombination, Cryptic sexuality, Mitochondrial introgression, Clonality

## Abstract

Many eukaryotic pathogenic microorganisms that were previously assumed to propagate clonally have retained cryptic sexual cycles. The principal reproductive mode of *Trypanosoma cruzi*, the aetiological agent of Chagas disease, remains a controversial topic. Despite the existence of two recent natural hybrid lineages, a pervasive view is that recombination has been restrained at an evolutionary scale and is of little epidemiological relevance to contemporary parasite populations. This article reviews the growing number of field studies which indicate that natural hybridization in *T. cruzi* may be frequent, non-obligatory and idiosyncratic; potentially involving independent exchange of kinetoplast and nuclear genetic material as well as canonical meiotic mechanisms. Together these observations now challenge the traditional paradigm of preponderate clonal evolution in *T. cruzi* and highlight the need for additional, intensive and appropriately sampled field surveys, complemented by high resolution, combined nuclear and mitochondrial population genetics analyses.

## Highlights

• The principal reproductive mode of *Trypanosoma cruzi* is controversial.
• Field studies indicate recombination is frequent, non-obligatory and idiosyncratic.
• These observations challenge the paradigm of clonal evolution in *T. cruzi*.

## Graphical abstract

**Figure:**
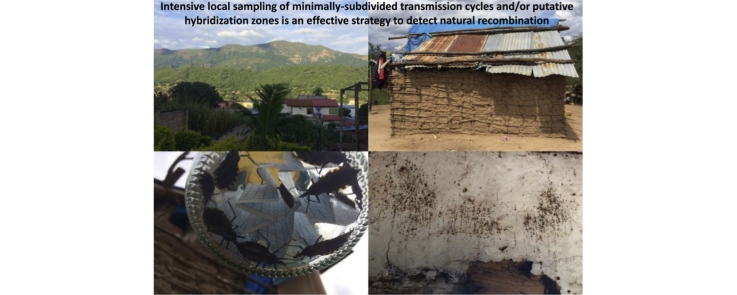

## 1 Introduction

The principal reproductive mode of a number of parasitic protozoan species is the subject of an enduring debate (Smith et al., 1993, Tibayrenc et al., 1986, Tibayrenc et al., 1990, Tibayrenc and Ayala, 1991, Tibayrenc and Ayala, 2012, Tibayrenc and Ayala, 2013, Tibayrenc and Ayala, 2014a, Tibayrenc and Ayala, 2014b, Ramírez and Llewellyn, 2014, Tomasini et al., 2014a, Tomasini et al., 2014b). At the two extremes are the preponderate clonal evolution (PCE) model, which suggests that genetic exchange is too infrequent to break the predominant pattern of clonality, such that only ‘restrained recombination’ occurs at an evolutionary scale (Tibayrenc and Ayala, 2012, Tibayrenc and Ayala, 2013, Tibayrenc and Ayala, 2014a, Tibayrenc and Ayala, 2014b), and the counter-proposition that hybridization is in fact pervasive, albeit challenging to detect, among some natural disease foci (Ramírez and Llewellyn, 2014).

*Trypanosoma cruzi*, the aetiological agent of Chagas disease, often fulfils some key assumptions of PCE, which have been cited as compelling evidence that it is essentially a clonal organism, namely strong linkage disequilibrium, deviations from Hardy–Weinberg allele frequencies and structuring of populations into stable, distinct genetic clades, or discrete typing units (DTUs) (Tibayrenc and Ayala, 2012, Tibayrenc and Ayala, 2013). *T. cruzi* isolates display remarkable genetic diversity, which is widely believed to contribute to the considerable biological, epidemiological and clinical variation observed among Chagas disease foci (Miles et al., 2009). Current international consensus recognizes a minimum of six genetic lineages or DTUs (TcI–TcVI), with distributions loosely defined by geography, ecology and transmission cycle (Zingales et al., 2009). Genotyping using an array of markers indicate DTUs TcI–TcIV form monophyletic clades and TcV and TcVI are recent, natural inter-lineage hybrids of TcII and TcIII (Machado and Ayala, 2001, Brisse et al., 2003, Lewis et al., 2011, Yeo et al., 2011). Molecular dating indicates that these hybrid lineages evolved recently, within the last 60,000 years (Lewis et al., 2011), possibly from human disruption of sylvatic transmission cycles in the Southern Cone of South America, suggesting there may still be a risk of genetic exchange driving the emergence of novel recombinants (Flores-López and Machado, 2011, Lewis et al., 2011). These hybrid DTUs circulate almost exclusively in domestic transmission cycles and are sympatric with severe clinical sequelae in southern endemic areas. However, the frequency of recombination occurring among natural *T. cruzi* populations, the precise cytological mechanisms underlying genetic exchange events and the effect of hybridization on parasite phenotype remain largely undefined.

A clear understanding of the impact of genetic exchange on the ecological and geographical distributions and clinical characteristics of *T. cruzi* strains is crucial to establish the epidemiological risk associated with recombinant genotypes and to reconcile the implications parasite hybridization has at both the generational and evolutionary scales. However, detecting genetic exchange among natural populations is inherently complicated by choice of samples and marker resolution, given that strains most likely to be recombining may be closely related and potentially indistinguishable.

## 2 Genetic exchange among *T. cruzi* field populations

With improved sampling strategies and the development of higher resolution nuclear and mitochondrial genotyping techniques (Llewellyn et al., 2009, Messenger et al., 2012, Messenger et al., 2015, Ramírez et al., 2012), a growing number of field studies now indicate that natural recombination in *T. cruzi* may be frequent, non-obligatory and idiosyncratic; potentially involving independent exchange of kinetoplast and nuclear genetic material as well as canonical meiotic mechanisms (Table 1).

At the inter-lineage level, DTUs TcV and TcVI are unequivocal hybrids, which resemble diploid, heterozygous Mendelian F1 progeny, sharing intact alleles from their parental progenitors (TcII and TcIII) (Machado and Ayala, 2001, Brisse et al., 2003, Barnabé et al., 2011, Lewis et al., 2009, Lewis et al., 2011, Yeo et al., 2011). The origin(s) of these hybrid DTUs is presently unresolved; it is unclear whether they arose from two independent genetic exchange events (De Freitas et al., 2006, Lewis et al., 2011) or a single incidence of hybridization followed by clonal divergence (Westenberger et al., 2005, Sturm and Campbell, 2010, Flores-López and Machado, 2011). The status of TcIII and TcIV as ancient recombinants of TcI and TcII (Westenberger et al., 2005), or sister groups of TcI (Tomasini and Diosque, 2015), is more contentious, and varies based on the array of nuclear loci (Westenberger et al., 2005, Tomasini and Diosque, 2015) or mitochondrial haplotypes examined (Lewis et al., 2011, Messenger et al., 2012). The evidence for any contemporary recombination between major DTUs is more limited and the genetic identity of each lineage appears largely preserved. However, it is not known to what extent this is maintained by genetic reproductive barriers between DTUs or ecological isolation, considering, for example, historical parents TcII and TcIII now circulate in almost completely separate transmission cycles.

**Fig. 1:**
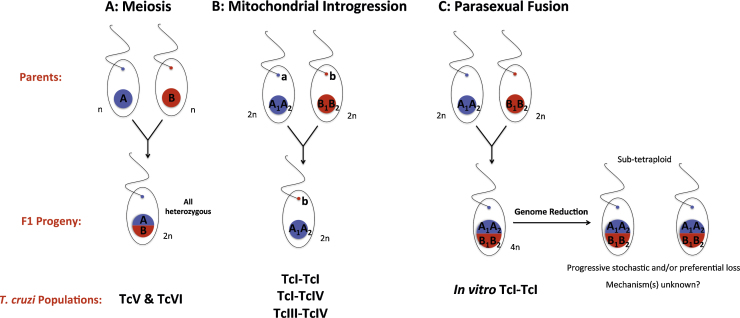
Putative cytological mechanisms and patterns of allele inheritance observed among natural field populations of *T. cruzi* (A and B) and during *in vitro* genetic exchange experiments (C).

At the intra-lineage level, genetic exchange is increasingly reported, particularly among TcI populations. It is unclear whether this is due to the examination of representatives from intensely sampled populations that are minimally-subdivided spatially and temporally, and therefore more likely to undergo hybridization, or if it truly reflects the analysis of strains that are more permissive to recombination (Prugnolle and De Meeus, 2010, Ramírez and Llewellyn, 2014). The underlying cytological mechanisms of natural intra-TcI recombination vary between studies and genetic markers used (Table 1 and Fig. 1).

In general, genetic exchange at the nuclear level has been demonstrated by Hardy–Weinberg allele frequencies, linkage equilibrium between loci, a lack of repeated multilocus genotypes (MLGs) (Barnabé et al., 2013, Baptista et al., 2014, Ocaña-Mayorga et al., 2010, Ramírez et al., 2013) and more rarely, excess heterozygosity (Messenger et al., 2015), all consistent with meiotic allele inheritance. In those studies that also examine kinetoplast DNA, mitochondrial introgression, evidenced by phylogenetic incongruence between nuclear and mitochondrial loci, is emerging as a common feature of natural transmission cycles especially within TcI populations (Lima et al., 2014, Messenger et al., 2012, Messenger et al., 2015, Ramírez et al., 2012, Zumaya-Estrada et al., 2012) but also historically, between major lineages (Lewis et al., 2011, Messenger et al., 2012, Barnabé and Breniere, 2012, Roellig et al., 2013) (Table 1 and Fig. 1). One explanation, given their crucial role in growth, development and metabolism, is that asymmetric mitochondrial introgression, may satisfy the elevated necessity to escape Muller's ratchet (the irreversible accumulation of deleterious mutations resulting from clonal reproduction) compared to the nuclear genome (Messenger et al., 2015, Neiman and Taylor, 2009, Ramírez and Llewellyn, 2014); others have attributed these observations to gross differences in evolutionary pressures and molecular clocks between non-coding microsatellites and coding maxicircle genes (Tibayrenc and Ayala, 2013). However, it is highly improbable that mutation rate variation could account for the observation of nearly identical nuclear genotypes with radically divergent mitochondrial genomes, particularly when putative donors and recipients are identified within the same population (Messenger et al., 2012, Ramírez et al., 2012).

Reciprocal nuclear recombination among parasite strains undergoing mitochondrial introgression has yet to be explicitly detected, which may support an asymmetric, cryptic hybridization mechanism, or perhaps more likely, reflect the minor amount of nuclear genetic information sampled (20% of the mitochondrial genome *vs*. <0.1% of the nuclear genome); without whole nuclear genome sequences for introgression hybrids and parental isolates, it is impossible to distinguish between these two hypotheses. However, by analogy to other medically important trypanosome species, the presence of alternate, covert sexual mechanisms within the same species is not entirely unexpected (Rougeron et al., 2009, Rougeron et al., 2011, Duffy et al., 2013, Hickman et al., 2013, Rogers et al., 2014, Ramírez and Llewellyn, 2014).

Interestingly, a recent study from Colombia identified biparental mitochondrial inheritance as a putative consequence of genetic exchange events (Ramírez et al., 2012). A mosaic maxicircle sequence was detected in a human TcI isolate and the presence of a recombination breakpoint confirmed by allele-specific PCR. Such a sequence is expected to arise following inter-molecular maxicircle recombination, which necessitates the inheritance of mixed mitochondrial complements. Uniparental inheritance of highly heteroplasmic maxicircles might present an indistinguishable scenario (if strains were characterized using basic dye-terminator sequencing) but reported mitochondrial heteroplasmy in *T. cruzi*, (examined using high coverage Illumina sequencing reads), is thus far low (Messenger et al., 2012). Parallel observations from experimental crosses of *Trypanosoma brucei brucei* (Gibson and Garside, 1990, Gibson et al., 2008), suggest that biparental mitochondrial inheritance might be a fundamental, as yet, uncharacterized, biological phenomenon in trypanosomatids.

## 3 Reconciling mechanisms of *T. cruzi* recombination *in vitro* and among natural populations

The generation of intra-TcI hybrids *in vitro* demonstrates that at least some *T. cruzi* strains have an extant capacity for genetic exchange (Gaunt et al., 2003). Putative parental isolates identified by Carrasco et al. (1996) were transformed with episomal recombinant plasmids containing either hygromycin B or neomycin resistance genes and co-passaged through *in vitro* (mammalian cell cultures) and *in vivo* (mice and triatomine bugs) cycles (Gaunt et al., 2003). Isolation of six clones by double drug selection from *in vitro* axenic cultures, and subsequent genetic characterization by MLEE, karyotyping, microsatellite genotyping and nucleotide sequencing of housekeeping genes, indicated that these intra-lineage recombinants inherited all parental alleles at most loci and one parental maxicircle genotype (Gaunt et al., 2003).

By analogy with *Candida albicans* (Bennett and Johnson, 2003, Forche et al., 2008), it was proposed that nuclear fusion had created a tetraploid intermediate, followed by homologous recombination, gradual genome erosion and reversion to aneuploidy (Fig. 1). FACs analysis of hybrid isolates reported a stable DNA content, on average, ∼69% higher than parental strains (Lewis et al., 2009); natural *T. cruzi* isolates are minimally diploid but overall genome size can vary by up to 48% between different DTUs (Lewis et al., 2009). Subsequent prolonged maintenance of the experimental hybrids in axenic cultures demonstrated a gradual, progressive decline in DNA content, with no evidence of any true meiotic reductive division; to date these strains remain sub-tetraploid (Lewis et al., 2010).

While this parasexual mechanism of genetic exchange has a precedent in fungal species, it is challenging to reconcile with both the predominant patterns of allele inheritance observed among natural *T. cruzi* populations (Table 1 and Fig. 1) as well as the conservation of meiosis-specific orthologues within the *T. cruzi* genome (Ramesh et al., 2005). A similar paradox exists in *T. b. brucei* where canonical meiotic recombination (Peacock et al., 2011), including the formation of haploid life cycle stages (Peacock et al., 2014), has been explicitly described, but is not the exclusive or obligate reproductive mechanism reported from transmission cycles (Balmer et al., 2011, Duffy et al., 2013). Likewise, experimental hybridization in *Leishmania* resembles meiosis with recurrent triploidy (Akopyants et al., 2009, Inbar et al., 2013), but inbreeding also appears frequent in nature (Calvo-Álvarez et al., 2014, Rougeron et al., 2009, Rougeron et al., 2011, Sterkers et al., 2011, Sterkers et al., 2014, Rogers et al., 2014).

## 4 Problems of detecting recombination among field populations

It is clear that to detect natural recombination, sample strategy, population allocation and marker choice are crucial for study design. Grouping of divergent non-recombining subgroups (in the case of *T. cruzi*, major DTUs) can inflate genetic linkage statistics and mask recombination events occurring between more closely related individuals (Smith et al., 1993). Recent observations of the Wahlund effect obscuring Hardy–Weinberg allele frequencies and linkage equilibrium within Brazilian TcII strains, caution the interpretation of statistics derived from inappropriately assigned parasite populations (Baptista et al., 2014). The use of multiple, different types of molecular markers (nuclear and mitochondrial, coding and non-coding) are required, in combination with targeted investigation of potential ‘hybridization’ zones, *i.e.* areas where recently diverged, genetically distinguishable subpopulations regularly interact (Messenger et al., 2015, Ramírez and Llewellyn, 2014). The value of such high-density sampling has already been demonstrated in defining the population structures of other trypanosomatid species, *e.g. Trypanosoma brucei gambiense* (Koffi et al., 2009), *Trypanosoma congolense* (Morrison et al., 2009), *Leishmania braziliensis* (Rougeron et al., 2009) and *Leishmania guyanensis* (Rougeron et al., 2011), including establishing putative levels of genetic exchange. However, the low circulating parasitaemia that defines chronic Chagas disease patients often prohibits parasite isolation, and thus many studies are overly reliant on historical collections of references isolates assembled over many years.

## 5 Implications of hybridization for parasite phenotype

Importantly, the effects of genetic exchange on *T. cruzi* phenotype are unknown. Hybrid vigour (heterosis) is a well-documented phenomenon among parasitic protozoa (Detwiler and Criscione, 2010). Observations of natural *Leishmania* hybrids indicate that genetic exchange can impact vector permissibility (Volf et al., 2007), increase (Akopyants et al., 2009) or alter (Cortes et al., 2012, Calvo-Álvarez et al., 2014) virulence, including the ability to disseminate and colonize visceral organs (Romano et al., 2014), as well as generate recombinant progeny that are capable of widespread clonal propagation (Schwenkenbecher et al., 2006, Nolder et al., 2007); a scenario reminiscent of the successful establishment of TcV and TcVI among domestic transmission cycles in the Southern Cone. Similarly, if mitochondrial introgression is exploitable as a putative mechanism of host range extension, hybrids might be expected to present higher mammalian infectivity and growth rates, especially in vectors (Messenger et al., 2015). The pathological implications of recombinant genotypes in human infections with regards to virulence, transmissibility and drug susceptibility warrant further investigation in conjunction with improved methods of identification and isolation of natural hybrids strains.

## 6 Conclusions

The majority of field studies now indicate that natural genetic exchange in *T. cruzi* is both contemporary and historical, responsible for shaping current parasite population structures, as well as the evolution of distinct *T. cruzi* DTUs. Together these observations challenge the traditional paradigm of PCE in *T. cruzi* and highlight the need for additional, intensive and appropriately sampled field surveys, complemented by high resolution, combined nuclear and mitochondrial population genetics analyses.

The precedent in experimental design established by such studies may represent the most promising intermediary in *T. cruzi* population genetics until imminently superseded by comparative population genomics.

## Figures and Tables

**Table 1:** Summary of field evidence of genetic exchange in *T. cruzi*.

*T. cruzi* population(s)	Transmission cycle, location	Type of genetic exchange	Genetic markers examined	Evidence of genetic exchange	Putative mechanism	Reference
TcI	Arboreal sylvatic, Bolivia	Intra-lineage	MLMT	- Mitochondrial introgression with no detectable nuclear involvement	Asymmetric mitochondrial introgression	Messenger et al. (2015)
			mtMLST	- Dissimilar heterozygosity estimates		
TcI	Bolivia	Inter-lineage	*GPI*^a^	- Mitochondrial introgression between TcI and TcIII/IV/V/VI with no detectable nuclear involvement	- Asymmetric mitochondrial introgression	Barnabé and Breniere (2012)
			*ND1*^b^			
TcI	Bolivia	Intra-lineage	MLMT	- H-W allele frequencies	- Meiotic	Barnabé et al. (2013)
				- Linkage equilibrium between loci		
				- Lack of repeated MLGs		
TcI	Arboreal sylvatic, Brazil	Intra-lineage	MLEE	- Putative homozygous parents and heterozygous progeny	- Meiotic	Carrasco et al. (1996)
			RAPD	- H–W phenotype frequencies		
TcII	Domestic, Brazil	Intra-lineage	MLMT	- H–W allele frequencies among local populations	- Meiotic	Baptista et al. (2014)
			*ND4*^b^, *ND7*^b^	- Linkage equilibrium between loci	- Asymmetric mitochondrial inheritance	
				- Independent inheritance of mitochondrial and nuclear genes		
TcI	Domestic, peridomestic, sylvatic, Colombia	Intra-lineage	MLMT	- Mitochondrial introgression with no detectable nuclear involvement	-Asymmetric mitochondrial introgression	Ramírez et al. (2012)
			mtMLST	- Recombinant mitochondrial genotype	- Biparental mitochondrial inheritance	
TcI	Domestic, peridomestic, sylvatic, Colombia	Intra-lineage	nMLST	- Linkage equilibrium between loci	- Meiotic	Ramírez et al. (2013)
				- Putative recombination breakpoints		
TcI	Domestic, Ecuador	Intra-lineage	MLMT	- H–W allele frequencies	- Meiotic	Ocaña-Mayorga et al. (2010)
				- Linkage equilibrium between loci		
				- Lack of repeated MLGs		
TcI and TcIII/IV	North America, Brazil, Bolivia	Inter-lineage	*GPI*^a^	- Mitochondrial introgression between TcIII and TcIV with no detectable nuclear involvement	- Asymmetric mitochondrial introgression	Lewis et al. (2011)
			*COII-ND1*^b^			
			MLMT			
TcI and TcIII/IV	North America, Venezuela, Argentina, Bolivia and Brazil	Inter- and intra-lineage	MLMT	- Mitochondrial introgression between TcI and TcIII/IV	- Asymmetric mitochondrial introgression	Messenger et al. (2012)
			mtMLST	- Intra-TcI mitochondrial introgression		
				- No detectable nuclear involvement		
TcI and TcIV	North America	Inter-lineage	*24Sα rRNA*^a^, *18S rRNA*^a^, *TcMSH2*^a^, *Tc55*^a^, *DHFR*-*TS*^a^, *COII*-*ND1*^b^	- Mitochondrial introgression between TcI and TcIV	- Asymmetric mitochondrial introgression	Roellig et al. (2013)

H–W: Hardy–Weinberg; MLEE: multilocus enzyme electrophoresis; MLG: multilocus genotype; MLMT: multilocus microsatellite typing; mtMLST: maxicircle multilocus sequence typing; nMLST: nuclear multilocus sequence typing; RAPD: random amplification of polymorphic DNA.

a Nuclear gene.

b Mitochondrial gene.
